# Waist-to-Height Ratio Is a Better Anthropometric Index than Waist Circumference and BMI in Predicting Metabolic Syndrome among Obese Mexican Adolescents

**DOI:** 10.1155/2014/195407

**Published:** 2014-12-09

**Authors:** Edel Rafael Rodea-Montero, María Lola Evia-Viscarra, Evelia Apolinar-Jiménez

**Affiliations:** ^1^Department of Research, Hospital Regional de Alta Especialidad del Bajío, Boulevard Milenio 130, San Carlos la Roncha, 37660 León, GTO, Mexico; ^2^Department of Pediatric Endocrinology, Hospital Regional de Alta Especialidad del Bajío, Boulevard Milenio 130, San Carlos la Roncha, 37660 León, GTO, Mexico

## Abstract

*Objective*. To identify the degree of association between anthropometric indices and components of metabolic syndrome (MS) and to determine optimal cut-off points of these indices for predicting MS in obese adolescents.* Methods*. A cross-sectional study with a sample of (*n* = 110) Mexican obese adolescents grouped by sex and the presence/absence of MS. BMI percentile, waist circumference (WC), and waist-to-height ratio (WHtR) were tested. ROC curves of the anthropometric indices were created to identify whether an index was a significant predictor of MS.* Results*. BMI percentile, WC, and WHtR were significantly correlated with systolic and diastolic blood pressure. As predictors of MS overall patients, the BMI percentile generated an area under curve (AUC) of 0.651 (*P* = 0.008), cut-off point above the 99th percentile. WC generated an AUC of 0.704 (*P* < 0.001), cut-off point of ≥90 cm. WHtR demonstrated an AUC of 0.652 (*P* = 0.008), cut-off point of 0.60. WHtR ≥0.62 and WHtR ≥0.61 generate AUC of 0.737 (*P* = 0.006) and AUC of 0.717 (*P* = 0.014) for predicting hypertension and insulin resistance, respectively, in females.* Conclusion*. WHtR is a better tool than WC and BMI for identifying cardiometabolic risk. The overall criterion (WHtR ≥ 0.6) could be appropriate for predicting MS in obese Mexican adolescents.

## 1. Introduction

The high prevalence of obesity in Mexico [1] leads to an increased incidence of type 2 diabetes mellitus (T2DM) [2] and the development of cardiovascular disease (CVD) in children [3].

Predominant abdominal adipose tissue distribution (central obesity) in adults and children is positively correlated with hypertension (HTN), dyslipidemia, and alterations in glucose metabolism, including insulin resistance (IR), glucose intolerance, and T2DM [4, 5].

In children (2 to 18 years of age), overweight (85th percentile ≤ body mass index (BMI) < 95th percentile) and obesity (BMI ≥ 95th percentile) are diagnosed according to BMI percentile [6]. However, it is debatable whether BMI and/or percentiles are accurate predictors of metabolic syndrome (MS) in children because BMI only reflects total obesity. Health risks are more associated with central obesity than total obesity. Based on this observation, many authors have proposed replacing BMI with waist circumference (WC) and/or waist-to-height ratio (WHtR) to determine the health risks of obesity and evaluating their use as MS screening tools [7, 8].

WHtR could be a better indicator of MS than WC because the latter index may change among children of the same age and sex in different height percentiles. Note that the height of children of the same age and sex can differ by up to 28 cm, which reflects a height variation between the 3rd and 97th percentiles. Therefore, WC could reflect different metabolic risks based on a child's height. In adults, studies have shown that people with the same WC but with shorter heights have a greater metabolic risk than taller people [9].

In general, pediatric anthropometric interpretation is difficult because the continuous growth process and adiposity rebound alter corporal composition. Moreover, ethnic-specific normality tables that permit assessments of sex and age percentile values are necessary to correctly interpret BMI. Interpretations and evaluations that help to identify obese children who are at greater risk of MS are complicated in the general practice setting. Practical tools are required to determine the presence of MS in a quick and more accurate manner in pediatric populations.

This study identified the degree of association between anthropometric indices (i.e., BMI percentile, WC, and WHtR) and MS components and determined the efficacy of each factor individually. The sensitivity, specificity, balanced accuracy, predictive positive value (PPV), predictive negative value (PNV), and optimal cut-off points of these indices were evaluated in terms of predicting MS in obese adolescents.

## 2. Materials, Subjects, and Methods

### 2.1. Subjects

Patients were diagnosed with obesity in primary and secondary health care services and then were referred and admitted to the Pediatric Obesity Tertiary Care Clinic at the Mexican Hospital Regional de Alta Especialidad del Bajío (HRAEB) in León city, Guanajuato state, between April 2008 and December 2012. We excluded children with chronic diseases, syndrome disorders, and endocrine obesity, as well as children who used any medication or other treatments.

A total of 110 adolescents (48 females and 62 males) ranging from 8 to 16 years of age were included in the analysis. Data from the “Metabolic Syndrome and Its Components among Obese (BMI ≥ 95th) Mexican Adolescents” protocol were used for this study [10]. The Research and Ethics Committees of the HRAEB evaluated and approved this study. Written informed consent was obtained from all patients.

### 2.2. Anthropometric Assessments

BMI was calculated as weight (kg) divided by height squared (m^2^). WC was measured at the end of normal expiration using a nonelastic tape measure (Seca, Hamburg, Germany) at the midpoint between the lower costal border and the iliac crest [11]. WC was measured to the nearest 0.1 cm, and WC percentiles were assessed using Mexican-Hispanic tables for children and adolescents [12]. WHtR was calculated by dividing WC by height [13].

### 2.3. Definition of Metabolic Syndrome

MS was diagnosed according to the National Cholesterol Education Program (ATP-III) criteria [14], as adapted by Cruz et al. [15], who standardized the absolute value of each MS component using the percentile value by age and sex. MS was defined as having at least three of the following abnormalities: WC (≥ the 90th percentile by age and sex), high triglycerides (TG) (≥ the 90th percentile by age and sex), low high-density lipoprotein cholesterol (HDL-C) (≤ the 10th percentile by age and sex), systolic or diastolic blood pressure (SBP and DBP, resp.) (≥ the 90th percentile by age, sex, and height) or undergoing antihypertensive treatments, and serum fasting glucose of at least 100 mg/dL.

### 2.4. Definition of Insulin Resistance

IR was considered when fasting insulin is greater than 121.98 pmol/L (17 *μ*UI/mL) (hyperinsulinism) [16] or when the homeostasis model assessment index (HOMA) was more than or equal to 3.16 [17]. HOMA was calculated as the concentration of fasting insulin (*μ*UI/mL) multiplied by the concentration of fasting plasma glucose (mmol/L)/22.5.

### 2.5. Statistical Analysis

Data were analyzed using R statistical software [18]. Descriptive statistics were determined for the patients' characteristics, grouped by the presence or absence of MS and compared using the Mann-Whitney *U* test. Anthropometric indices of interest (i.e., BMI percentile, WC, and WHtR) in the study population were grouped by sex and the presence or absence of MS and then calculated and tested using the Kruskal-Wallis test.

Spearman's correlation coefficients between the anthropometric indices and MS components were calculated and tested.

Receiver operating characteristic (ROC) curves of the anthropometric indices (overall and by sex) were created to identify whether an index was a significant predictor of MS, and the area under the corresponding curve (AUC) was used to evaluate the predictive efficiency of each index. Additionally WHtR was evaluated as a single predictor of each non-WC component of MS and IR. Different optimal cut-off points in the selected indices and the corresponding sensitivity, specificity, balanced accuracy, PPV, and PNV were estimated. The optimal cut-off point in each case was calculated as the minimum value of the square root of [(1 − sensitivity)^2^ + (1 − specificity)^2^], and greater accuracy is reflected by a smaller distance to the point (0,1).

The sample size allowed the detection of a ≥10% difference in any assessment (with type I error *α* = 0.05 and type II error *β* = 0.80). In all cases, 95% confidence intervals were constructed, and a *α* = 0.05 level of significance was used for all tests.

## 3. Results

The mean age ± standard deviation for all patients was 11.55 ± 2.02 years (range: 8.11–15.97 years).

The prevalence of MS was 62% overall. Considering each component of MS, the prevalence in the sample was high TG 85%, low HDL-C 60%, abdominal obesity 88%, hyperglycemia 5%, and HTN 35%. Additionally the prevalence of hyperinsulinism was 33% overall (35% in female and 31% in male) and the prevalence of HOMA-IR was 38% overall (40% in female and 37% in male).

Patients were divided into two groups based on the presence or absence of MS. Descriptive statistics for the patients' characteristics are shown in Table 1. Patients' ages and Tanner stages were similar in both groups (*P* = 0.455 and *P* = 0.399, resp.). In terms of body composition, weight and height were significantly greater in the children with MS (*P* = 0.003 and *P* = 0.007, resp.). BMI and BMI percentile were significantly greater in the children with MS (*P* = 0.010 and *P* = 0.008, resp.). A statistically significant difference in variable WHtR was detected (*P* = 0.008); the patients with MS demonstrated higher values. No significant between-group differences were detected for insulin levels and HOMA (*P* = 0.662 and *P* = 0.638, resp.).

According to the MS components and based on the intergroup comparison, statistically significant differences were detected for TG (*P* = 0.007) and WC (*P* < 0.001); the patients with MS had higher values. HDL-C (*P* < 0.001) levels were significantly lower in the patients with MS. The glucose levels did not differ between the groups (*P* = 0.530). Higher SBP and DBP levels were detected in the patients with MS (*P* < 0.001 in both cases).

Table 2 illustrates the anthropometric indices of interest (i.e., BMI percentile, WC, and WHtR) in the study population (grouped by sex and the presence or absence of MS). In terms of BMI percentile (*P* = 0.054), there were no differences between groups. Comparisons between WC and WHtR showed significant differences (*P* = 0.003 and *P* = 0.025, resp.); both values were greater in the patients with MS.

Spearman's correlation coefficients (*r*) between the anthropometric indices (BMI percentile, WC, and WHtR) and MS components overall are shown in Table 3. Significant correlations were detected. BMI percentile and WHtR were significantly correlated with WC (*r* = 0.61, *P* < 0.001 and *r* = 0.78, *P* < 0.001), SBP (*r* = 0.39, *P* < 0.001 and *r* = 0.19, *P* = 0.043), and DBP (*r* = 0.32, *P* < 0.001 and *r* = 0.25, *P* = 0.009), respectively. WC was only correlated with blood pressure (SBP and DBP) (*r* = 0.41, *P* < 0.001 and *r* = 0.34, *P* < 0.001). None of the anthropometric indices were significantly correlated with TG, HDL-C, and fasting glucose.

Table 4 shows the areas under the ROC curves (AUC), with 95% confidence intervals, for the selected anthropometric indices, with cut-off points for sensitivity, specificity, balanced accuracy, PPV, and PNV for a MS diagnosis. Some significant AUCs were estimated and are described below. In men, the BMI percentile demonstrated an AUC of 0.677 (*P* = 0.021), with a cut-off point above the 98th percentile; this estimated cut-off point was included in the criterion for predicting MS in males. WC in males and females generated an AUC of 0.696 (*P* = 0.011) and an AUC of 0.708 (*P* = 0.016), respectively, as well as cut-off points >90 cm and >95 cm, respectively. Moreover, WC considered that all patients generated an AUC of 0.704 (*P* < 0.001) and a cut-off point of ≥90 cm to predict MS in obese adolescents, with 67.6% sensitivity, 66.7% specificity, 67.2% balanced efficiency, 76.7% PPV, and 56.0% PNV. We estimated that WHtR in females provided an AUC of 0.682 (*P* = 0.034) and a cut-off point of ≥0.60; in males, the AUC was 0.627 (*P* = 0.098, which was not significant but apparent), with a cut-off point of ≥0.63.

**Figure 1:**
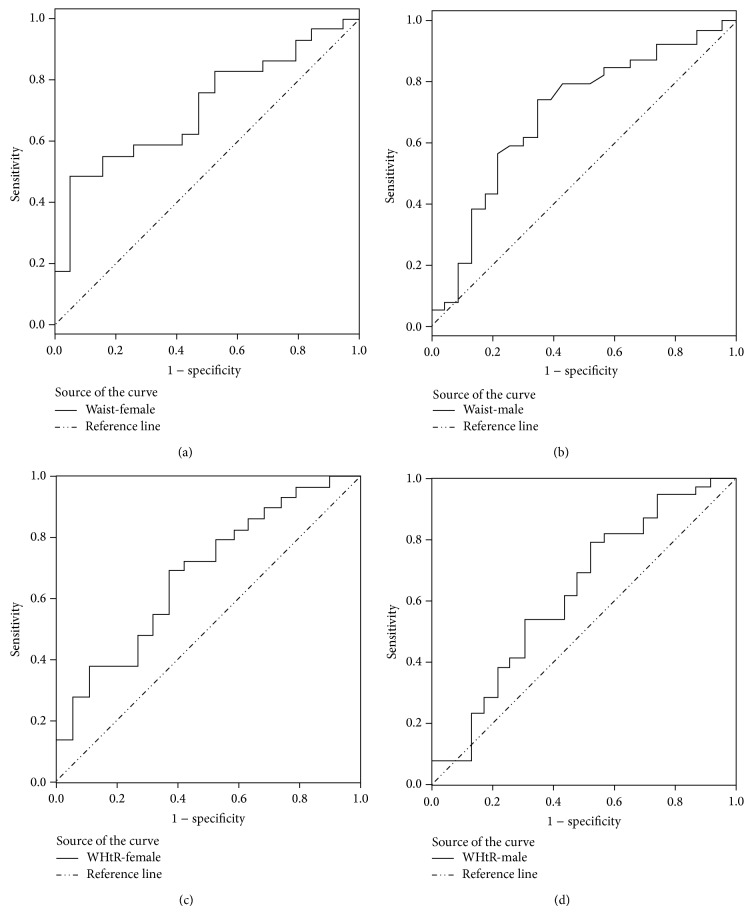
Receiver operating characteristic (ROC) curves for predicting metabolic syndrome by sex: (a) waist-female, (b) waist-male, (c) WHtR-female, and (d) WHtR-male.

Finally, in terms of WHtR for all patients, we estimated an AUC of 0.652 (*P* = 0.008) and an optimal cut-off point of 0.60, which provided a WHtR criterion of ≥0.60 for predicting MS in obese adolescents, with 69.1% sensitivity, 57.1% specificity, 63.1% balanced efficiency, 72.3% PPV, and 53.3% PNV. Detailed graphs of the WC and WHtR ROC curves for predicting MS by sex are shown in Figure 1.

Table 5 shows the evaluation of WHtR as a single predictor of non-WC components of MS, hyperinsulinism, and HOMA-IR. We detected statistically significant AUCs for HTN and IR in females; in the case of HTN, WHtR in females provided an AUC of 0.737 (*P* = 0.006) and a cut-off point of ≥0.62. According to hyperinsulinism, WHtR in females provided an AUC of 0.717 (*P* = 0.014) and a cut-off point of ≥0.61, and considering HOMA-IR, WHtR in females provided an AUC of 0.713 (*P* = 0.013) and a cut-off point of ≥0.61.

## 4. Discussion

As members of the Mexican obesogenic environment, children are often overfed, which can result in a variety of health consequences. The estimated prevalence of MS in Mexican obese children (BMI ≥ 95th) is 62–72% [10, 19], which is high compared to other countries [20, 21]. In this study, TG, SBP, and DBP levels were higher, and HDL-C levels were lower in obese children with MS compared to obese children without MS. No significant between-group differences in insulin and HOMA were detected, but the children with MS demonstrated values indicating IR, which could be associated with increased anthropometric assessments, such as height, BMI, BMI percentile, WC, and WHtR.

Previous studies showed that WC and WHtR were most useful in identifying cardiometabolic risks in normal-weight and overweight children and adults [22–24]. Our study indicated that not all obese children have metabolic risks, and we consider BMI to be an insufficient anthropometric tool for detecting MS.

WC and WHtR assessments, which adjust for the variability of height between children of the same age, more precisely reflected the percentage of intra-abdominal adipose tissue (IAAT, intra-abdominal visceral fat) than BMI [25]. Greater central or visceral adipose tissue distribution in obese children is clearly a factor responsible for metabolic comorbidities (IR, HTN, and dyslipidemia) and the development of cardiovascular complications [5, 26, 27].

In children and adolescents, comparative image studies (directly measuring IAAT) and anthropometric indices (BMI, WC, and WHtR) demonstrate that metabolic alterations and cardiovascular risk factors are correlated more with WC and WHtR than BMI. Teixeira et al. used dual-energy X-ray absorptiometry to evaluate fat distribution in children, and they concluded that WHtR was positively correlated with cholesterol, TG, LDL-C, and apolipoprotein B and negatively correlated with HDL-C. Moreover, the authors observed that BMI was negatively correlated with HDL-C but BMI did not correlate with other metabolic factors [28]. Brambilla et al. used magnetic resonance imaging to show that BMI was correlated more with subcutaneous adipose tissue than with abdominal visceral adipose tissue [29]. These direct methods suggest that BMI does not appear to be the better anthropometric index for predicting the risk of developing metabolic complications because BMI does not directly reflect the percentage of IAAT.

The increase in height during growth spurts in childhood and adolescence indicates that WC could decrease to reflect a lower correlation with cardiovascular risk factors. Tybor et al. suggest that WHtR maintains a considerable and variable residual correlation with height during childhood and adolescence. This residual correlation may affect how this measurement of central adiposity relates to risk factors of interest [30]. In our study, obese patients with MS demonstrated greater height, WC, and WHtR assessments than obese patients without MS. This observation indicates that growth in obese adolescents does not decrease WC if their lifestyles (diet and physical activity) are not modified.

The duration of central obesity is an independent risk factor for the development of T2DM, and elevated childhood BMI has repeatedly been associated with increased risks of cardiovascular disease, left ventricular hypertrophy, and mortality in early adulthood [31]. The estimated probability that an obese child will become an obese adult is 80% [32], which suggests that overfeeding in the early stages of life implies a high cardiovascular risk to our pediatric population.

One disadvantage of using the WHtR index is that the cut-off points for defining obesity and/or central adiposity excess are under discussion, and, currently, there is no consensus. Initial studies of WHtR in children proposed an arbitrary WHtR cut-off point of ≥0.5, which was extrapolated from adult studies [23, 33, 34].

Klünder-Klünder and Flores-Huerta observed that, in Mexican populations, children of both sexes with WC in the 75th percentile or greater have a WHtR of ≥0.5; however, cardiometabolic risk factors were not evaluated in their descriptive study [12]. Similarly, Nambiar et al. proposed 0.5 as a cut-off point for WHtR to define obesity in a pediatric population [35]. This same cut-off point has been evaluated to predict HTN in obese and nonobese children [36].

Our results suggest the following optimal cut-off points for the anthropometric indices used to predict MS among obese Mexican adolescents: BMI ≥ the 99th percentile or severe obesity [6], WC ≥90 cm for males and ≥95 cm for females (absolute values), and WHtR ≥0.6 for females and ≥0.63 for males. The WHtR cut-off point for males is higher than that for females; some authors have detected this difference and suggest that it may be attributed to a wider range in body weight in male subjects than female subjects [37].

The overall patient analysis (males and females) generated a WHtR cut-off point of ≥0.6, and this value facilitated the use of a unique value for predicting MS among obese Mexican adolescents. This same cut-off point has been proposed by Khoury et al. to identify cardiovascular risk in overweight and obese children [16]. WHtR is recognized as a rapid and effective global indicator of the health risks associated with obesity, with the following advantages: it is more sensitive, less expensive, and easier to measure than BMI, and it is applicable to different ethnic groups [7]. However, BMI has been characterized as an estimate of the percentage of body fat, and BMI percentiles in children continue to define overweight and obesity worldwide [6]. Moreover, BMI is a moderately sensitive and specific indicator of excess adiposity among children [38].

When we evaluated WHtR as a predictor of non-WC components of MS and IR overall and by sex, we identified that WHtR is a single predictor of HTN and IR in females, with WHtR cut-off points of ≥0.62 and ≥0.61, respectively. IR is also a well-known contributor of HTN in adolescents [39]. The study of Kruger et al. identified a cut-off point of WHtR ≥0.41 for predicting HTN and HOMA-IR in African adolescents [40] and Beck et al. in Brazilian adolescents identified a cut-off point of WHtR ≥0.48 for HTN in females [41] but both studies include obese and nonobese adolescents and the cut-off points described are lower than the international proposed cut-off value of WHtR ≥0.5 which permits identifying obesity. The importance of determining a simple tool (WHtR) for predicting HTN in obese female adolescents will be helpful to prevent HTN complications: future altering cardiac output, cardiac systolic and diastolic function, and renal-pressure natriuresis [42].

Obesity and the associated IR are considered as the main risk factor for developing intolerance glucose and T2DM regardless of genetic predisposition [43]. As a response of the alarming increase of T2DM in youth recent studies focus on the association between WHtR and IR (hyperinsulinism or HOMA-IR) in adolescents [40] but there are few studies that propose a cut-off point of WHtR for predicting IR; besides WC and WHtR were found to be strongly associated with IR [44]. Our proposed cut-off of WHtR advantages the prediction of IR in obese Mexican female adolescents that helps to implement preventive measures against the development of T2DM [39].

This study has some limitations. First, it was a cross-sectional study; therefore, causality cannot be inferred. Second, some critics might suggest that the generalizability to other ethnic groups is limited. Moreover, the identified cut-off points used to predict MS among obese adolescents are consistent with the estimated cut-off points in other ethnic groups. The strengths of our study include the comparison of various anthropometric indices among only obese adolescents, the identification of WHtR as a good predictor of MS in our study population, and the identification of WHtR as a good predictor of HTN and IR in females.

## 5. Conclusion

This study demonstrated that WHtR is a better tool than WC and BMI for identifying cardiometabolic risk in obese Mexican adolescents. Our results indicated that the overall criterion (WHtR ≥ 0.6) could be appropriate for predicting MS in obese Mexican adolescents. Additionally the criteria (WHtR ≥ 0.62 and WHtR ≥ 0.61) could be considered for predicting HTN and IR, respectively, in obese Mexican female adolescents. Faced with an obesity epidemic in the Mexican population, the use of proposed cut-off points for WHtR will help us to identify and send obese patients with MS to specialized centers. We estimate that 38% of obese (BMI in the 95th percentile or greater) children do not have MS, and these children could be treated at primary and secondary health care centers. Our proposal would help to provide timely care to obese adolescents with MS and to avoid the overpopulation of specialized (tertiary) care centers.

## Figures and Tables

**Table 1:** Characteristics of the study population, grouped by the presence or absence of metabolic syndrome.

	Without MS (−) (*n* = 42)	With MS (+) (*n* = 68)	Intergroup comparison^a^
Clinical, anthropometric, and biochemical			
Age, years	11.41 (2.2)	11.63 (1.91)	*P* = 0.455
Tanner stage, median	2	3	*P* = 0.399
Weight, kg	63.38 (16.75)	74.5 (19.57)	*P* = 0.003^b^
Height, cm	146.27 (10.72)	152.12 (11.63)	*P* = 0.007^b^
BMI, kg/m^2^	28.94 (5.03)	31.62 (5.24)	*P* = 0.010^b^
BMI percentile	98.22 (0.99)	98.66 (1.04)	*P* = 0.008^b^
WHtR	0.61 (0.06)	0.64 (0.06)	*P* = 0.008^b^
Fasting insulin, pmol/L	96.72 (78.06)	133.96 (147.88)	*P* = 0.662
HOMA	2.88 (2.37)	4.08 (4.62)	*P* = 0.638
Components of MS			
Triglycerides, mmol/L	1.69 (0.75)	2.16 (0.96)	*P* = 0.007^b^
HDL-C, mmol/L	1.15 (0.26)	0.88 (0.17)	*P* < 0.001^b^
Waist, cm	88.88 (10.12)	96.77 (11.22)	*P* < 0.001^b^
Fasting glucose, mmol/L	4.79 (0.30)	4.86 (0.42)	*P* = 0.530
SBP, mmHg	105.81 (8.88)	114.38 (10.01)	*P* < 0.001^b^
DBP, mmHg	61.14 (8.76)	68.21 (9.04)	*P* < 0.001^b^

Unless otherwise indicated, the values are given as the mean (standard deviation).

^a^Mann-Whitney *U* test.

^b^Significant *P* values.

**Table 2:** Anthropometric indices of the study population, grouped by sex and the presence or absence of metabolic syndrome.

	Overall (*n* = 110)	Female (*n* = 48)		Male (*n* = 62)		Intergroup comparison^a^
		Without MS (−) (*n* = 19)	With MS (+) (*n* = 29)	Without MS (−) (*n* = 23)	With MS (+) (*n* = 39)	
BMI percentile	98.49 (1.04)	98.27 (0.95)	98.54 (1.18)	98.18 (1.05)	98.76 (0.93)	*P* = 0.054
Waist, cm	93.76 (11.43)	87.35 (8.84)	95.49 (11.15)	90.15 (11.1)	97.73 (11.31)	*P* = 0.003^b^
WHtR	0.63 (0.06)	0.59 (0.04)	0.63 (0.05)	0.62 (0.07)	0.64 (0.06)	*P* = 0.025^b^

Unless otherwise indicated, values are given as the mean (standard deviation).

^a^Kruskal-Wallis test with 3 degrees of freedom.

^b^Significant *P* values.

**Table 3:** Spearman's correlation coefficients (*r*) between the anthropometric indices and components of MS.

	BMI percentile		Waist		WHtR	
	*r*	Significance	*r*	Significance	*r*	Significance
Triglycerides	0.04	*P* = 0.671	0.07	*P* = 0.465	0.01	*P* = 0.917
HDL-C	−0.02	*P* = 0.842	−0.15	*P* = 0.122	−0.09	*P* = 0.349
Waist	**0.61**	*P* < 0.001^a^	—	—	**0.78**	*P* < 0.001^a^
Fasting glucose	0.16	*P* = 0.102	0.01	*P* = 0.893	0.17	*P* = 0.081
SBP	**0.39**	*P* < 0.001^a^	**0.41**	*P* < 0.001^a^	**0.19**	*P* = 0.043^a^
DBP	**0.32**	*P* < 0.001^a^	**0.34**	*P* < 0.001^a^	**0.25**	*P* = 0.009^a^

In all cases, 110 subjects (48 females and 62 males) were considered.

^a^Significant *P* values.

**Table 4:** Area under the ROC curves and 95% confidence intervals for the selected anthropometric indices, with cut-offs for the sensitivity, specificity, balanced accuracy, PPV, and PNV of an MS diagnosis.

Variable	Area under the ROC curve (95% C.I.)	Significance^a^	Optimal cut-off point^b^	Sensitivity	Specificity	Balanced accuracy	PPV^c^	PNV^d^
BMI percentile								
Overall	0.651 (0.547–0.755)	*P* = 0.008^e^	**99**	61.8%	66.7%	64.3%	75.0%	51.9%
Female	0.606 (0.441–0.771)	*P* = 0.217	99	65.5%	68.4%	67.0%	76.0%	56.5%
Male	0.677 (0.540–0.814)	*P* = 0.021^e^	**98**	76.9%	56.5%	66.7%	75.0%	59.1%
WC (cm)								
Overall	0.704 (0.604–0.804)	*P* < 0.001^e^	**90**	67.6%	66.7%	67.2%	76.7%	56.0%
Female	0.708 (0.561–0.854)	*P* = 0.016^e^	**95**	55.2%	84.2%	69.7%	84.2%	55.2%
Male	0.696 (0.556–0.836)	*P* = 0.011^e^	**90**	74.4%	65.2%	69.8%	78.4%	60.0%
WHtR								
Overall	0.652 (0.544–0.759)	*P* = 0.008^e^	**0.60**	69.1%	57.1%	63.1%	72.3%	53.3%
Female	0.682 (0.528–0.836)	*P* = 0.034^e^	**0.60**	69.0%	63.2%	66.1%	74.1%	57.1%
Male	0.627 (0.477–0.776)	*P* = 0.098	0.63	53.8%	69.6%	61.7%	75.0%	47.1%

In all cases, 110 subjects (48 females and 62 males) were considered.

^a^Null hypothesis: area = 0.5.

^b^Positive if assessment is more than or equal to the optimal cut-off point; it was calculated as the minimum value of the square root of the following: [(1 − sensitivity)^2^ + (1 − specificity)^2^], and greater accuracy is reflected by a smaller distance to point (0, 1) in the ROC curve.

^c^PPV: predictive positive value.

^d^PNV: predictive negative value.

^e^Significant *P* values.

**Table 5:** Area under the ROC curves and 95% confidence intervals for WHtR, with cut-offs for the sensitivity, specificity, balanced accuracy, PPV, and PNV of individual MS components, hyperinsulinism, and HOMA-IR diagnosis.

Variable	Area under the ROC curve (95% C.I.)	Significance^a^	Optimal cut-off point^b^	Sensitivity	Specificity	Balanced accuracy	PPV^c^	PNV^d^
WHtR								
High TG overall	0.600 (0.441–0.760)	*P* = 0.200	0.60	62.8%	62.5%	62.7%	90.8%	22.2%
High TG female	0.581 (0.251–0.912)	*P* = 0.555	0.58	76.7%	60.0%	68.4%	94.3%	23.1%
High TG male	0.622 (0.441–0.803)	*P* = 0.207	0.60	66.7%	63.6%	65.2%	89.5%	29.2%
Low HDL-C overall	0.501 (0.389–0.613)	*P* = 0.985	0.63	43.9%	63.6%	53.8%	64.4%	43.1%
Low HDL-C female	0.613 (0.451–0.774)	*P* = 0.188	0.60	64.3%	55.0%	59.7%	66.7%	52.4%
Low HDL-C male	0.416 (0.269–0.562)	*P* = 0.266	0.63	42.1%	54.2%	48.2%	59.3%	37.1%
Hyperglycemia overall	0.606 (0.335–0.877)	*P* = 0.426	0.65	60.0%	69.5%	64.8%	8.6%	97.3%
Hyperglycemia female	0.689 (0.388–0.990)	*P* = 0.277	0.65	66.7%	82.2%	74.5%	20.0%	97.4%
Hyperglycemia male	0.567 (0.009–1.000)	*P* = 0.750	0.76	50.0%	93.3%	71.7%	20.0%	98.3%
Hypertension overall	0.601 (0.491–0.711)	*P* = 0.081	0.63	51.3%	64.8%	58.1%	44.4%	70.8%
Hypertension female	0.737 (0.584–0.891)	*P* = 0.006^e^	**0.62**	66.7%	73.3%	70.0%	60.0%	78.6%
Hypertension male	0.512 (0.359–0.665)	*P* = 0.876	0.66	38.1%	68.3%	53.2%	38.1%	68.3%
Hyperinsulinism overall	0.599 (0.490–0.780)	*P* = 0.093	0.61	69.4%	52.7%	61.1%	41.7%	78.0%
Hyperinsulinism female	0.717 (0.569–0.865)	*P* = 0.014^e^	**0.61**	70.6%	64.5%	67.6%	52.2%	80.0%
Hyperinsulinism male	0.509 (0.358–0.660)	*P* = 0.909	0.61	68.4%	44.2%	56.3%	35.1%	76.0%
Insulin resistance (HOMA) overall	0.566 (0.459–0.673)	*P* = 0.245	0.61	64.3%	51.5%	57.9%	45.0%	70.0%
Insulin resistance (HOMA) female	0.713 (0.567–0.859)	*P* = 0.013^e^	**0.61**	68.4%	65.5%	67.0%	56.5%	76.0%
Insulin resistance (HOMA) male	0.457 (0.312–0.602)	*P* = 0.575	0.59	73.9%	28.2%	51.1%	37.8%	64.7%

In all cases, 110 subjects (48 females and 62 males) were considered.

^a^Null hypothesis: area = 0.5.

^b^Positive if assessment is more than or equal to the optimal cut-off point; it was calculated as the minimum value of the square root of [(1 − sensitivity)^2^ + (1 − specificity)^2^], and greater accuracy is reflected by a smaller distance to point (0, 1) in the ROC curve.

^c^PPV: predictive positive value.

^d^PNV: predictive negative value.

^e^Significant *P* values.
